# Protocol of the adaptive study of IL-2 dose frequency on regulatory T cells in type 1 diabetes (DILfrequency): a mechanistic, non-randomised, repeat dose, open-label, response-adaptive study

**DOI:** 10.1136/bmjopen-2015-009799

**Published:** 2015-12-08

**Authors:** Lucy A Truman, Marcin L Pekalski, Paula Kareclas, Marina Evangelou, Neil M Walker, James Howlett, Adrian P Mander, Jane Kennet, Linda S Wicker, Simon Bond, John A Todd, Frank Waldron-Lynch

**Affiliations:** 1 JDRF/Wellcome Trust Diabetes and Inflammation Laboratory, National Institute for Health Research Cambridge Biomedical Research Centre, Cambridge Institute for Medical Research, University of Cambridge, Cambridge Biomedical Campus, University of Cambridge, Cambridge, UK; 2 National Institute for Health Research Cambridge Clinical Trials Unit, Cambridge University Hospitals NHS Foundation Trust, Cambridge Biomedical Campus, Cambridge, UK; 3 MRC Biostatistics Unit Hub for Trials Methodology Research, Cambridge Institute of Public Health, Cambridge, UK; 4 Department of Mathematics, Imperial College, London, UK

**Keywords:** DIABETES & ENDOCRINOLOGY, IMMUNOLOGY, GENETICS

## Abstract

**Introduction:**

Type 1 diabetes (T1D) is caused by autoimmune destruction of the insulin-producing β cells in the pancreatic islets, leading to insulinopenia and hyperglycaemia. Genetic analyses indicate that alterations of the interleukin-2 (IL-2) pathway mediating immune activation and tolerance predispose to T1D, specifically the polymorphic expression of the IL-2 receptor-α chain (CD25) on T lymphocytes. Replacement of physiological doses of IL-2 could restore self-tolerance and prevent further autoimmunity by enhancing the function of CD4^+^ T regulatory cells (Tregs) to limit the activation of auto reactive T effector cells (Teffs). In this experimental medicine study, we use an adaptive trial design to determine the optimal dosing regimen for IL-2 to improve Treg function while limiting activation of Teffs in participants with T1D.

**Methods and analysis:**

The *Ad*aptive study of *IL-*2 dose *frequency* on Tregs in type 1 diabetes(DILfrequency) is a mechanistic, non-randomised, repeat dose open-label, response-adaptive study of 36 participants with T1D. The objective is to establish the optimal dose and frequency of ultra-low dose IL-2: to increase Treg frequency within the physiological range, to increase CD25 expression on Tregs, without increasing CD4^+^ Teffs. DILfrequency has an initial learning phase where 12 participants are allocated to six different doses and frequencies followed by an interim statistical analysis. After analysis of the learning phase, the Dose and Frequency Committee will select the optimal targets for Treg frequency, Treg CD25 expression and Teff frequency. Three groups of eight participants will be treated consecutively in the confirming phase. Each dose and frequency selected will be based on statistical analysis of all data collected from the previous groups.

**Ethics:**

Ethical approval for DILfrequency was granted on 12 August 2014.

**Results:**

The results of this study will be reported, through peer-reviewed journals, conference presentations and an internal organisational report.

**Trial registration numbers:**

NCT02265809, ISRCTN40319192, CRN17571.

**Strengths and limitations of this study**

- This is an experimental medicine response-adaptive study that is statistically designed to analyse three coprimary immunological end points to efficiently determine the optimal dose-frequency ultra-low dose interleukin-2 (IL-2) in type 1 diabetes (T1D).
- The study will investigate the effects of repeated doses of ultra-low dose IL-2 on the immune system of participants with T1D.
- The mechanism of action of ultra-low dose IL-2 will be characterised in T1D prior to considering larger phase II/III clinical trials.
- The study is not designed to test the efficacy of ultra-low dose IL-2 treatment in T1D.

## Introduction

Type 1 diabetes (T1D) is caused by a loss of tolerance of the immune system (autoimmunity) to the body's own insulin-producing β cells of the pancreas, leading to their dysfunction and/or destruction resulting in insulin deficiency and hyperglycaemia.^1^ Autoreactive effector T lymphocytes (Teffs) are central to disease pathogenesis and it is thought that many cases of T1D are caused by poor regulation of Teffs by CD4^+^ FOXP3^+^ T regulatory cells (Tregs).^2^ The degree of β-cell destruction and insulin deficiency depends on the age of patients at diagnosis and the duration of their disease. Children diagnosed under age five usually progress rapidly and completely lose their ability to make insulin, whereas those diagnosed as adolescents or adults may preserve a low level of insulin production for decades.^3^ Enhancing β-cell survival and function is a key goal of T1D immunotherapy because preservation of even small amounts of endogenous insulin production can reduce the requirement for exogenous insulin, a potentially dangerous drug. In addition preservation of a limited β-cell function can improve glucose metabolism, reduce harmful glycosylation of proteins in the body, protect against hypoglycaemia and prevent microvascular complications such as retinopathy, nephropathy and neuropathy in the long term.4–6a

The interleukin-2 (IL-2) pathway is one of the most important genetically validated pathways with therapeutic relevance to T1D.^7^ IL-2 signalling via the high-affinity, heterotrimeric IL-2 receptor which comprises CD25 (α chain), CD122 (β) and CD132 (γ), is essential for the development and maintenance of Tregs that sustain self-tolerance and prevent autoimmunity.^8^ Genome-wide association studies have identified several genes in the IL-2 pathway (eg, *IL2RA* encoding CD25, *PTPN2, IL2-IL21* and *BACH2*) that are associated with an increased risk of developing T1D.^9^ Rare monogenic disorders in either *FOXP3* (a transcription factor that drives CD25 expression and the suppressive function of Tregs) or mutations in the CD25 gene (*IL2RA*) itself, cause severe autoimmune syndromes including T1D.^10^ 10a Analysis and phenotyping of T cells from patients and controls with variations in *IL2RA* showed that reduced CD25 expression on T cells is associated with susceptibility to T1D.^11–13^ Other defects in the IL-2 signalling pathway in Tregs affecting pSTAT5^11^ ^14^ and FOXP3^15^ can also reduce self-tolerance to β cells. Tregs are preferentially activated by IL-2 because they constitutively express 10-fold higher levels of the heterotrimeric high-affinity IL-2 receptor than Teffs. The higher sensitivity of Tregs for IL-2 provides a potential ‘therapeutic window’ where it might be possible to administer ultra-low doses of IL-2 in order to promote Treg function without stimulating a potentially unfavourable Teff response. Ultra-low dose IL-2 is amenable to pharmaceutical intervention owing to the availability of human recombinant IL-2, (Proleukin, also called Aldesleukin, manufactured by Novartis Pharmaceuticals UK, Limited; https://www.medicines.org.uk/emc/medicine/19322) which has extensive human safety data available. Proleukin has been used for the treatment of cancer and more recently, in trials for the treatment of the inflammatory disorders graft-versus-host-disease^16 17^ and hepatitis C induced vasculitis.^18^

We are implementing an innovative, experimental medicine strategy to deliver immunotherapy that systematically targets the key aetiological pathways in T1D.^7^ ^19^ ^20^ DILfrequency and its forerunner, ‘A*d*aptive study of *IL*-2 dose on regulatory T cells in *t*ype *1 d*iabetes’ (DILT1D)^20^ ^21^ are specifically designed to analyse the effects of Proleukin on the human peripheral immune system in blood to establish the dose and frequency of administration required to preferentially enhance Tregs over Teffs. DILT1D was designed to estimate the single dose of Proleukin required to increase the frequency of Tregs by a minimum of 10% and a maximum of 20% over baseline. DILT1D also included a detailed, mechanistic analysis of the effects of Proleukin on the whole immune system, in particular any activation of the Teff arm of the immune system was investigated. DILT1D is completed and the analyses are ongoing with the results being prepared for publication. We have used the available data to determine the initial doses to be used in the learning phase of DILfrequency.

The goal of DILfrequency is to find the optimal dose and frequency of subcutaneous Proleukin that specifically increases Treg frequency, and the amount of CD25 on Tregs, without expanding the Teff population in participants with T1D. There is an urgent need for this information because previous trials of Proleukin in inflammatory diseases^16–18^ and in T1D^22–26^ have used relatively high doses of Proleukin. These high doses of Proleukin that were administered in these studies as in induction protocols have a greater potential to activate Teffs. In addition, the very large increases of Tregs observed in these trials were far beyond the physiological range and could lead to immunosuppression and increased susceptibility to infections^16–18^ ^22–26^. In contrast, our aim is to deliver optimal amounts of Proleukin at a precisely determined frequency that is immunomodulatory to T cells to restore the T1D immune system to a healthy homeostatic Teff-Treg relationship. Previously, the frequency of Proleukin dosing has been empirically derived from clinical experience of high-dose Proleukin as immunotherapy for metastatic renal cell carcinoma^27^ and HIV infection.^28^ We now know that these high doses of Proleukin given in ‘on then off’ treatment cycles are more suitable for cancer treatment to activate Teffs and are not optimal for preserving insulin secretion and treating T1D. Results from a recent trial^29^ giving T1D participants Rapamycin with 4.5×10^6^ IU of Proleukin three times a week for a month was terminated prematurely because β-cell function was impaired. Rapamycin is used routinely for immunosuppression in pancreatic islet transplantation and therefore, the observed decline in β-cell function could be due to the high dose of Proleukin activating Teffs. Alternatively, Proleukin may have altered the effects of Rapamycin on β cells.

In DILT1D and DILfrequency we are taking a different approach: by using all of the data generated in the studies together with statistical modelling we aim to find the optimal dose and dosing-schedule for the future administration of Proleukin to attempt to preserve β-cell function in newly diagnosed participants with T1D.^21^

## Methods

### Study design

DILfrequency is a mechanistic, non-randomised, repeat dose, open-label, response-adaptive study of Proleukin, recombinant IL-2 (Aldesleukin; Marketing Authorisation Holder: Novartis Pharmaceuticals UK Limited)*.* This is a single centre study located at the National Institute for Health Research/Wellcome Trust Cambridge Clinical Research Facility, Addenbrooke's Hospital and the University of Cambridge Clinical School. The co-ordination of the study will be carried out by the National Institute for Health Research Cambridge Clinical Trials Unit, Cambridge University Hospitals NHS Foundation Trust.

**Figure 1:**
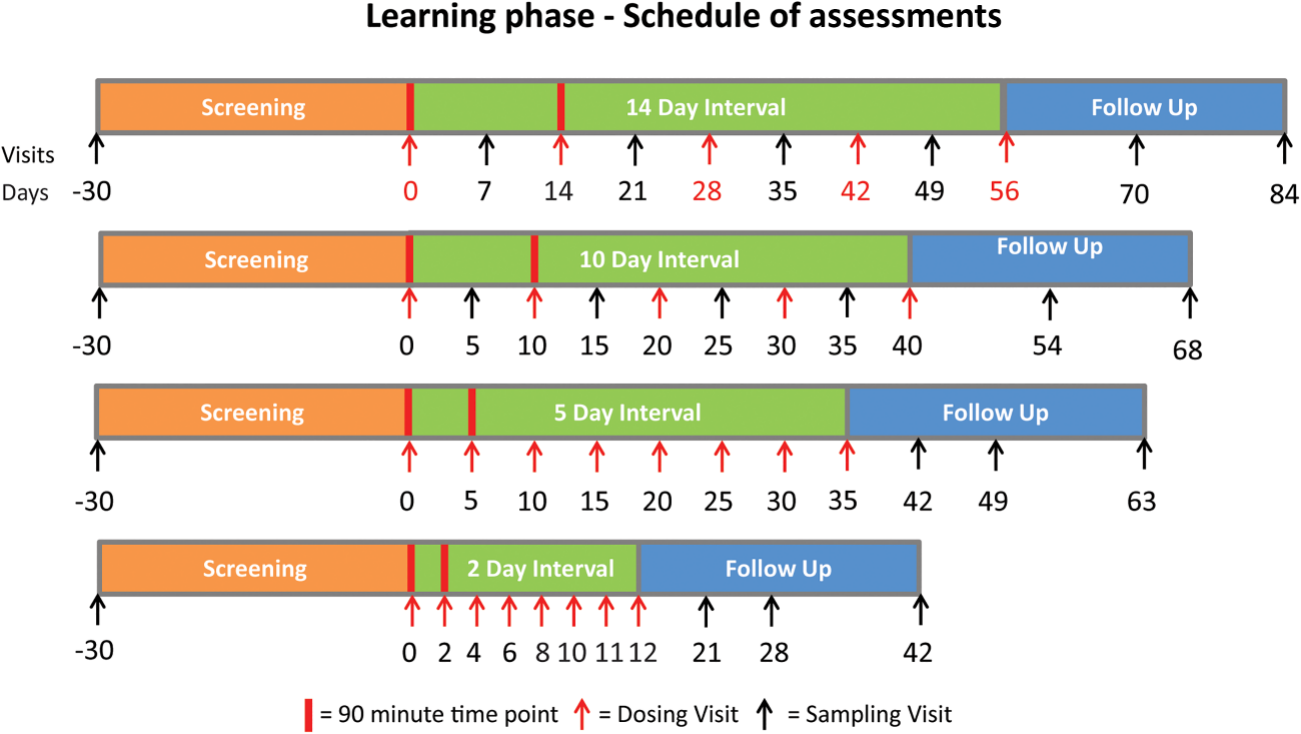
Study design of the learning phase of DILfrequency. The study has 12 visits and starts with a screening visit followed by a treatment period of 10 visits and a follow-up visit that will be carried out approximately 4 weeks after the final dose of Proleukin. Twelve participants will be allocated in the learning phase to two different doses and four frequencies of administration of Proleukin to measure the change from baseline of CD4^+^ T regulatory cells (Tregs), CD4^+^ T effectors and CD25 expression on Tregs during treatment with ultra-low dose interleukin-2. At the first two dosing visits, 90 min time points are measured to access early immune activation and the effects of repeat dosing on these events.

Thirty-six participants with T1D will be included in the study. Participants will be enrolled into DILfrequency for approximately 10–18 weeks depending on the duration of their treatment. Every participant has 12 visits beginning with a screening visit followed by a treatment period of 10 visits and a follow-up visit approximately 4 weeks after the final dose of drug (figure 1). The trial starts with a learning phase followed by three groups of participants in a tripartite confirming phase. The groups are sequentially analysed, so that data from all of the preceding participants informs the next group. The expected duration of DILfrequency is 2 years and the clinical part of the study will end when the last participant attends the last follow-up visit.

### Study participants: consent procedure and recruitment

Eligible participants will have a history of T1D and be less than 60 months from diagnosis (box 1). Potential participants will be ineligible if they have a history of severe organ dysfunction, malignancy, active clinical infection, active autoimmune hyperthyroid or hypothyroidism and a donation of more than 500 mL of blood in the 2 months prior to treatment box 2. Eligible potential participants that are interested in the study will be provided with a patient information sheet and a consent form to review. They will then be contacted by the study team to determine if they remain interested in participating in the study or have any further queries. Interested potential participants will be invited to attend for an appointment where the chief investigator (CI) or his delegate will discuss the study with the participant, who will then provide written informed consent before undergoing any trial-related procedures.

Box 1 **Inclusion criteria**

- Type 1 diabetes
- 18–70 years of age
- Duration of diabetes less than 60 months from diagnosis
- Written and informed consent to participate

To maximise participation in DILfrequency we will deploy the active internet recruitment strategy that was successfully established for DILT1D.^30^ Details of DILfrequency will be posted on http://www.clinical-trials-type1-diabetes.com/ with updates for participant engagement posted on https://www.facebook.com/ClinicalTrialsType1Diabetes and tweeted on https://twitter.com/t1diabetestrial. Interested potential participants may then directly contact the study team to discuss the study. Treating physicians, diabetes nurses and research nurses may identify potential participants and with permission, forward the patient's contact details to the study team. The study team will also contact potential participants who have already registered an interest in taking part in a clinical trial by enrolling in the ADDRESS-2 register,^31^ the D-GAP^32^ or DILT1D^20^ studies. A formal recruitment analysis has been specified as an exploratory end point in DILfrequency using the methodology developed for DILT1D.^30^

### Coprimary end points

The coprimary end points will be calculated from the change from baseline measurements and the average of the last three trough values where: the three coprimary end points are the frequency of Tregs, CD25 expression on Tregs and the frequency of Teffs. Baseline measurements are taken at visit two prior to drug administration and trough values are those obtained immediately before drug is administered representing the constant, lowest level observed assuming that a steady state has been achieved.

Box 2 **Eligibility criteria**

- Hypersensitivity to Proleukin or any excipients
- History of severe cardiac disease
- History of malignancy within the past 5 years (with the exception of localised carcinoma of the skin that had been resected for cure or cervical carcinoma in situ)
- History or current use of immunosuppressive agents or steroids
- History of unstable diabetes with recurrent hypoglycaemia
- History of live vaccination 2 weeks prior to first treatment
- Active autoimmune hyperthyroidism or hypothyroidism
- Active clinical infection
- Major pre-existing organ dysfunction or previous organ allograft
- Females who are pregnant, lactating or intend to get pregnant during the study
- Males who intend to father a pregnancy during the study
- Donation of more than 500 mL of blood within 2 months prior to Proleukin administration
- Participation in a previous therapeutic clinical trial within 2 months prior to Proleukin administration
- Abnormal ECG
- Abnormal full-blood count,
- Chronic renal failure (stage 3, 4 or 5) and/or evidence of severely impaired liver function
- Alanine transaminase or aspartate transaminase >3× upper limit of normal (ULN) at screening;
- Alkaline phosphatase and bilirubin 2× ULN at screening (isolated bilirubin >2× ULN is acceptable if bilirubin is fractionated and direct bilirubin <35% is measured).

**Figure 2:**
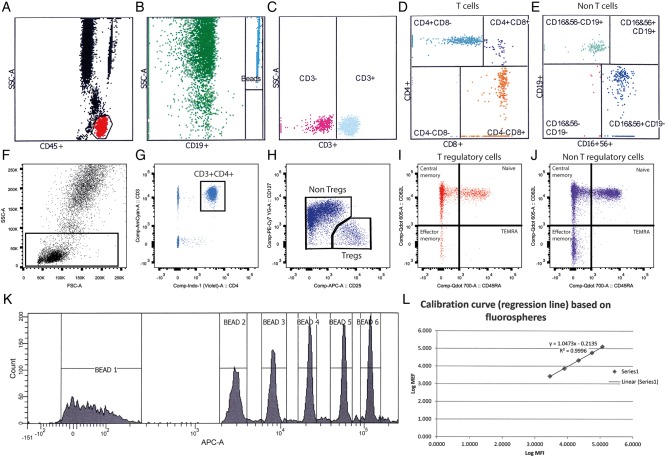
Clinical fluorescence-activated cell sorting (FACS) assays on whole blood to measure absolute lymphocyte counts, proportions of lymphocytes and CD25 on T regulatory cells. Fluorescently labelled beads are added to whole blood and analysed to accurately count the absolute number of lymphocytes (A), beads (B), CD3^+^ T cells (C) CD4^+^ and CD8^+^ T cells (D) CD19^+^ B cells and CD19^−^, CD16^+^, CD56^+^ NK cells as a percentage of all lymphocytes (E). In a parallel whole blood FACS assay, a lymphocyte gate is drawn to include all events (F) and doublets are excluded (not shown). The CD3^+^, CD4^+^ T-cell gate excludes CD8^+^ T cells and B cells (G). CD127^−^, CD25^+^ T regulatory cells (Tregs) are separated from non-Tregs since the non-Tregs are heterogeneous for CD127 and CD25 and this percentage is used to calculate the absolute Treg count out of CD4^+^ (H), naïve, effector memory, central memory and total effector memory CD45 RA^+^(TEMRA) Tregs (I) and non-Tregs (J) are measured according to CD62L and CD45RA expression, as shown. A cocktail of six standardised beads labelled with different amounts of fluorescent allophycocyanin (APC) are measured by FACS daily to accurately measure CD25-APC on the surface of Tregs (K) and a standard curve plotted (L). The mean fluorescence intensity of CD25^+^ on Tregs can be accurately read from the curve, minimising interassay variation.

The number of Tregs and Teffs and CD25 expression on Tregs are defined within the CD3^+^CD4^+^ fluorescence-activated cell sorting (FACS) gate (figure 2).

- Tregs % CD25^high^CD127^low^ within the CD3^+^CD4^+^ gate
- CD25 expression on Tregs is defined as the mean fluorescence intensity (MFI) of CD25 allophycocyanin (APC) within the Treg (CD3^+^CD4^+^CD25^high^CD127^low^) gate.
- Teff populations (non-Tregs) account for all the other CD3^+^CD4^+^ cells that are not defined as Tregs within the CD3^+^CD4^+^ gate:
  Effector memory % CD45RA^−^CD62L^−^
  Central memory % CD45RA^−^CD62L^+^
  Naïve Teffs % CD45RA^+^CD62L^+^
  Effector memory RA+ (TEMRA) CD45RA^+^CD62L^−^
  Central memory %+ Effector memory % = Total Memory effectors %
  Change in the ratio Naïve effectors %:Total Memory effectors % defines the Teff primary end point.

The FACS assay used to measure the three primary end points will be carried out according to good laboratory practice at the Clinical Immunology Laboratory, Department of Immunology, Addenbrooke's Hospital, Cambridge, which is an MHRA accredited laboratory for Good Clinical Practice.

### Secondary end points

- Change in Treg frequency, phenotype and proliferation will be measured by FACS at baseline, during and following treatment with Proleukin.
- Change in Teff frequency, phenotype and proliferation will be measured by FACS at baseline, during and following treatment with Proleukin.
- Change in natural killer (NK) cell frequency, phenotype and proliferation will be measured by FACS at baseline, during and following treatment with Proleukin.
- Change in B lymphocyte cell frequency, phenotype and proliferation will be measured by FACS at baseline, during and following treatment with Proleukin.
- Change in T and NK cell intracellular signalling will be measured by FACS at baseline, during and following treatment with Proleukin.
- Change in full-blood count will be measured by automatic analyser at baseline, during and following treatment with Proleukin.
- Change in plasma/serum levels of IL-2, IL-6, IL-10 and tumour necrosis factor α will be measured by ELISA.
- Change in metabolic control will be measured by self-monitoring of blood glucose and insulin use; glycated haemoglobin (HbA1c), C-peptide and autoantibody status will be clinically measured and recorded.

The values for the end points will be measured during the treatment period and followed for 4 weeks after the last dose for each participant.

### Exploratory end points

- Genotype of T1D-associated loci in DNA extracted from blood of participants.
- Gene expression and epigenetic analysis of purified lymphocyte subsets.
- IL-2 sensitivity of Tregs, Teffs and NK subsets in cryopreserved peripheral blood mononuclear cells (PBMCs).
- Treg suppression and T effector proliferation assays on cryopreserved PBMCs.
- Antigen specific T cell assays on cryopreserved PBMCs.
- Sysmex^®^ analysis of whole blood.
- Serum/plasma levels of cytokines, soluble receptors and inflammatory markers.
- Serum/plasma and cellular metabolites.
- Recruitment analysis.

### Safety assessments

Safety and tolerability will be accessed by clinical history, physical examination, temperature, blood pressure, heart rate, 12-lead ECGs, clinical laboratory tests and adverse event recording.

### Treatment assignment and monitoring of administration

At the start of the study in the learning phase the first 12 participants will be allocated the doses and frequencies (table 1) that are at the extremes of the available combinations. Group 1 will start treatment first, followed sequentially by groups 2–4. A multivariate model of the joint distribution of the end points (Tregs, Teffs and CD25 on Tregs) will be developed as a function of dose and frequency and the target ranges for each of the three primary end points will be determined at the first interim analysis meeting. The probability that each dose/dose-frequency achieves its target will be estimated. The Dose and Frequency Committee (DFC) will determine the rules that govern the optimal dose and dose frequency of Proleukin to be given to participants in the next group. Based on ongoing analyses of the results from our single dose study of Proleukin, DILT1D, the maximum dose of Proleukin that will be assigned is 0.6×10^6^ IU/m^2^/day because doses above this level may activate Teffs. The doses and frequencies selected for participants in the confirming phase are allowed to be different to those used in the learning phase.

**Table 1:** DILfrequency treatment assignment

Learning phase				
Group	Dose interval	Total number of doses	Number of participants	
			Minimal dose	Maximal dose
1	14th day	5	2	2
2	10th day	5	2	
3	5th day	8		2
4	2nd day	8	2	2

Confirming phase		
Group	Dose/interval	Number of participants
5	Determined from analysis of learning phase data	8
6	Determined from analysis of data from all previous participants	8
7	Determined from analysis of data from all previous participants	8

The first dose of Proleukin will be administered subcutaneously by participants at the study site following instruction by an appropriately trained delegated member of the trial team. Further doses may then be self-administered by participants or an appropriately qualified member of the study team depending on participant preference. The date and time of each dose administered will be recorded in the source documents. In the community, the date and time of each dose administered will be recorded in the participant's study diary that will become part of the source documents. The study diary, injection sites and count of unused medication will be carried out at each study visit while on treatment to confirm compliance.

### Dose and Frequency Committee and study governance

The role of the DFC is to provide decisions regarding the choice of dose and frequency of Proleukin to administer to participants in the confirming phases (groups 5, 6 and 7). After each group has completed its dosing schedule the data with be extracted from the DILfrequency OpenClinica database. The data will be delivered to the DFC within two working days of that date. The data will include assessments of safety, dose and dose-frequencies assigned for all participants to that time point and Teff and Treg frequencies and CD25 expression on Tregs, collated from all visits.

The DFC will make the following decisions:

- A safety assessment will be made by a clinician;
- Determine if steady state has been achieved for each participant;
- Define the numerical targets for Treg increase, increased CD25 expression on Tregs and a minimal increase in Teffs at the first DFC;
- Define the dose and dose-frequencies for the next group of participants.

The DILfrequency statisticians are responsible for preparing the DFC report that will include:

- Plots of all the participant profiles (Treg, CD25, Teff response vs time);
- Plots of the sequence of doses and dose intervals;
- Scatter plots of the coprimary end points (average of the trough values) versus dose and dose interval.
- Scatter plots of the coprimary end point adjusted for covariates versus dose and dose interval with superimposed fitted linear regression models with 95% confidence bands reporting:
  Log-likelihood;
  Raw output from statistical packages;
  Residual plots of each model fitted.

The details of the report may evolve as the study progresses. The quorate DFC will be comprised of a statistician, physician (chair and CI) and scientist, each member has a single vote and decisions can be reached by a majority. The Trial Steering Committee (TSC), comprising: an independent chairperson; the CI; the Cambridge Clinical Trials Unit, trial coordinator; a scientist and statistician with relevant experience, may be asked to review split decisions. Prior to the DFC meetings, any protocol violations will be reported to the statisticians and analyses will include the whole population.

### Statistical methods

This study is an exploratory study that is not designed to formally test a hypothesis in a confirmatory fashion and so a formal power calculation is inappropriate. The sample size of 36 is achievable within the proposed time scale, given the recruitment rates we obtained at our study site for DILT1D study.^20^ ^21^ Statistical simulations have estimated that 36 participants will provide valuable information under scenarios that represent a scientifically plausible and clinically relevant relationship between dose/frequency and the three coprimary end points. There will be four groups of participants in total, and with the longest interval being 14 days with a maximum duration of treatment of 98 days before an interim analysis. This will allow time to observe all four groups within the desired total study period of 2 years. Statistical analysis will be performed after each of the four phases of DILfrequency and the accuracy of target prediction is expected to increase after each round of analysis.

A tri-variate normal distribution will be assumed for the three coprimary end points (Teff, Treg and CD25 on Tregs). A model linking dose and dose-frequency to the mean and covariance parameters will be developed. At the first interim analysis the DFC will fix the numerical values that define the target range of the primary end point. The probability of each dose/frequency laying within the target range set at the first interim analysis will be tabulated along with the parameter estimates for all models considered, using SE and 95% CIs. The plausibility of the modelling assumptions generated by the study statistician will be reviewed by the DFC. In subsequent cohorts the choice of dose/frequencies to allocate will be determined by:

- Setting a minimum estimated probability of reaching the target to allow a dose/frequency to be considered eligible for further allocation;
- The ranking of the probabilities if multiple dose/frequencies are eligible.

If no dose/frequencies from those observed are eligible for allocation then other dose/frequencies may be considered. The decision guidelines are to be finalised prior to the first interim analysis based on simulations of the operating characteristics for a variety of scenarios. The simulations will be updated if any of the prior assumptions are not close to the interim values, this includes whether a more complex model is required.

Summary statistics will be provided for all coprimary and secondary end points, broken down by dose/frequency. Any such summary must use a minimum of five participants to ensure statistical plausibility, and any smaller subgroups will be merged to achieve the minimum size. Continuous variables will be summarised using mean, SD, median, maximum and minimum; categorical and binary variables will be summarised using frequency tables reporting in an ‘x/n (p %)’ format. A formal statistical analysis plan will be finalised in advance of the final data analysis.

## Discussion

DILfrequency will be an open-label, sequential study designed to estimate the optimal frequency and dose of administration of ultra-low doses of Proleukin that is required to maintain an increase in Tregs and an increased Treg response, that is CD25 expression, without expanding Teffs in patients with T1D. The secondary objective is to characterise the effects of repeated doses of Proleukin on the immune system. For example, a subset of NK cells express CD25 and are highly responsive to IL-2^33^ ^34^ and we aim to analyse the effects of Proleukin administration on this subpopulation of cells in some detail. Our approach contrasts with a traditional randomised, double-blind placebo-controlled trial design used in T1D immunotherapy trials to date. In a traditional trial design drug mechanisms, molecular events and biomarkers can only be related to the outcome once the results are un-blinded at the end of the trial. Moreover, previous trials have empirically estimated the dose and frequency of agents, based on chemotherapy regimens for cancer and this may have contributed to the limited efficacy of Proleukin observed in these trials.^22^ ^25^ ^35^ ^36^ In our view, these analyses should be completed before the planning and design of further clinical testing of Proleukin for T1D therapy in phase II and III trials.

The selection of primary end points for the DILfrequency study has been informed by our experience in DILT1D in which a dose-dependent increase in Tregs was observed and an increase in CD25 expression on Tregs was found to be a sensitive and reproducible marker of Proleukin administration (unpublished results). It will be important to establish that repeat dosing in DILfrequency does not lead to an increase in Teffs. Therefore, Teffs are the third of the coprimary end points that will be measured in DILfrequency. In addition to the three coprimary end points, this study will define the cellular outcome of frequent administration of Proleukin by detailed immunophenotyping, genetic, epigenetic and transcriptional analyses of peripheral blood subsets from participants before, during and after Proleukin treatment. These exploratory assays will be performed in order to define the mechanism of drug action and to understand the effect of repeated dosing of Proleukin on biomarkers.

DILfrequency challenges any pretrial assumptions about what the ‘optimal’ dose-frequency should be, by continuously adapting the dose-frequency after each interim analysis. The adaptive protocol outlined here allows for the most efficient derivation of the optimum dose-frequency and potentially, fewer participants are required compared to an equivalent fixed trial design. Furthermore, the DILfrequency design has the flexibility to adapt to a wider range of dose frequencies of Proleukin than could be pragmatically investigated in a single fixed-dose study. The higher number of participants at or near the optimal dose and dose-frequency in DILfrequency improves the statistical power for analysing mechanistic and biomarker data in addition to the information gained by obtaining multiple measures from each participant over time.

We propose that our approach to drug development can be adopted widely across a range of disorders and that it is an important foundation to a future of precision medicine.
